# Type 2 diabetes and health care costs in Latin America: exploring the need for greater preventive medicine

**DOI:** 10.1186/s12916-014-0136-z

**Published:** 2014-08-19

**Authors:** Armando Arredondo

**Affiliations:** National Institute of Public Health-Mexico, Visiting Professor in sabbatical period, School of Public Health of the University of Montreal, 7101 avenue du Parc, 3e étage, Bureau 3074-5, Montreal, Qébec H3N 1X9 Canada

**Keywords:** Diabetes, Chronic diseases, Universal coverage, Epidemiological transition, Economic burden, Latin American countries

## Abstract

**Background:**

Despite advances in medicine, health systems in Latin America are not coping with the challenges of chronic diseases. Incidence of disease and the economic burdens as a consequence have both increased in recent years. We have chosen Type 2 diabetes as an example to highlight the challenges posed by chronic diseases, in terms of the epidemiological transition and the economic burden of the demand for services to treat such problems.

**Discussion:**

Current health systems are not prepared to respond in a comprehensive manner to all phases of the natural history of the disease. There are new models of universal coverage, but resources and models of care are focused on programs aimed at healing/rehabilitation, and very sparsely at detection/prevention.

**Summary:**

In this scenario, chronic problems have alarmingly increased direct costs (medical care) and indirect costs (temporary disability, permanent disability and premature mortality). If more resources are not assigned to preventive medicine, these trends, in addition to not meeting the needs of the population, will financially collapse health systems and the patients’ pockets. This Opinion piece outlines some possible changes that can be implemented to better prepare the health services in Latin American countries.

## Background

In recent decades, the epidemiological transition that middle-income countries have been facing creates great challenges for health systems. Indeed, this phenomenon of transition has been characterized by a significant increase in the prevalence of chronic diseases (Type 2 diabetes, renal disease, hypertension, and so on) while communicable diseases are still present (gastroenteritis, respiratory infections, vector-borne diseases, and so on). In this context, health systems have a demand for increasingly diversified services and more financial resources to meet new demands (for chronic diseases) which compete widely for the resources allocated to service demands for communicable diseases.

As an example of chronic diseases we can consider Type 2 diabetes, which has become one of the top diseases contributing to increased morbidity and mortality in most countries of the world, generating great challenges for medicine and public health. Indeed, in the latest report from the World Atlas of Diabetes registry for 2010, there are 285 million adults with diabetes [1]. This number can be expected to continue to increase globally due to an aging population, growth in population size, urbanization and a high prevalence of obesity and sedentary lifestyles.

As part of the epidemiological transition, the latest Global Burden of Disease (GBD) recently reported that by 2010, Type 2 diabetes [2], as a tracer of the global epidemiological transition, is one of the biggest challenges facing health systems and societies today.

## Discussion

### The challenges facing existing Latin American health systems

In most Latin American countries (LACs) (mainly in Mexico, Argentina, Chile and Brazil), the prevalence of infectious diseases still presents a burden, and in addition there has been accelerated growth of chronic diseases, such as diabetes and hypertension [3,4]. This goes against the classic model of epidemiological transition with significant presence of communicable diseases and non-communicable diseases. According to the GBD report, the epidemiological burden of Type 2 diabetes will have the greatest impact on the health systems and society in LACs (see Box 1).

**Figure 1:**
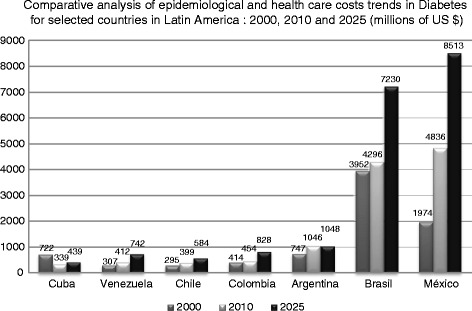
**Epidemiological and economic burden of diabetes in Latin America.** Comparing 2010 versus 2025, epidemiological trends of changes in diabetes in Latin America will have a high impact in economic terms. If no significant changes are made in the current health care model in these countries, health systems will face a constant and growing increase in the financial resources required to meet the demand for health services, particularly in countries like Brazil, Argentina and Mexico.

With regard to Type 2 diabetes, in economic terms, the epidemiological transition phenomenon represents a heavy burden, both in direct costs to the health system and society and indirect costs attributable to premature mortality and temporary and permanent disability attributable to complications of diabetes [5,6]. Indeed, the costs observed in the group of countries selected for this essay (Cuba, Venezuela, Colombia, Chile, Argentina, Brazil and Mexico) [7–9], show increasing trends if current epidemiological conditions and current models of care are maintained. This is especially true for Mexico, Argentina and Brazil (Figure 1).

On the other hand, in Latin America and the Caribbean, many people with Type 2 diabetes have limited access to health care due to the associated high costs and because public health services are not available to all people; this means indirect costs may exceed direct health care costs. This situation generates catastrophic health expenditures, particularly for the population that is not covered by the health system.

In terms of the response of the health system in recent years, health systems in most LACs have undertaken adjustments, changes or reforms in national health programs, trying to meet the goal of universal health insurance. Longer life expectancy and fewer families impoverished by health problems are some of the results of the recent adoption of universal health insurance in these countries.

In terms of universal health coverage, we could say that in the last decade LACs have undergone a great change. Indeed, we have generated new schemes of universal health insurance, with significant progress in improving access to health services. The approaches and results vary from country to country, but the evidence indicates that one of the shared priorities is to ensure that quality care reaches the poorest and the population not covered by the health system [10,11].

Indeed, since 2005, the ‘Argentine Maternal-Child Health Insurance Program’ has helped introduce historical changes in the health system in Argentina. More than one million pregnant women and children who lacked health insurance, now have basic health insurance and access to services for maternal and child health needs [12]. In the case of Brazil, the Unified Health System funded by taxes, modernized the Brazilian health system, creating a national coordinated service that the entire population can access. They have invested heavily in a primary care strategy referred to as ‘Family Health’, in which health professionals, community leaders and heads of families work together in primary care actions. This strategy has been the vehicle used to carry out major reforms allowing families greater access to health care through home visits and activities to improve health.

In Chile, the ‘Social Health Insurance’ program ensures nearly universal health coverage for the country’s 17 million inhabitants. Since 2005, all Chileans have had access to a basic package that guarantees treatment of 80 health problems such as renal disease, retinopathy, hypertension, and so on, establishing maximum waiting times for treatment and discretionary spending. In Colombia, the right to health was established in its constitution in 1991; 20 years later, access to health services has improved considerably thanks to a national system of ‘subsidized health insurance’. By making the central government responsible for providing health services, the subsidized regime has significantly strengthened the right to health. In the case of México, the Popular Health Insurance, covering 50 million people, promotes access to health care for all those who lack social security. At the heart of the 2003 health reform, this coverage package includes more than 200 primary and secondary treatments for the entire population, including health services for both communicable and chronic degenerative diseases.

Despite advances in coverage under schemes of ‘Universal Health Insurance’ in all these countries, the epidemiological and economic burdens of problems such as hypertension and Type 2 diabetes are far from resolved, and prevalence continues to increase incrementally. If major changes are not implemented into the relevant health care models, there will be significant challenges in dealing with the rising costs as shown in Figure 1.

In this context, the problem is that even with more coverage and access to health care, the model remains the same as when it originated in the 1940s. Indeed, the health systems in LACs, are based on a fragmented model with several institutions providing health care for people in the formal economy (social security institutes) versus institutions for population in the informal economy (ministries of health) [13] . These institutions provide health services based on a model of care with a biomedical curative approach [14,15]. The resulting benefits of this model, for chronic problems such as Type 2 diabetes, have not been favorable.

In summary, despite the increased coverage schemes of universal health insurance, the current characteristics of the health care models and the expected epidemiological changes for LACs represent catastrophic expenditures for health systems and patients. Moreover, the high financial burden for governments and societies of these countries will have to face internal competition in the allocation of resources to other diseases.

### Management of chronic illness requires significant adjustments to health care practice

As noted in the Background, models of health care in LACs, from their origins to date, have been structured and organized primarily to meet the health needs of patients with communicable diseases. Moreover, these models have focused their attention on healing actions, while neglecting early detection and prevention. In the case of chronic diseases, this is particularly relevant, especially because it is the ‘silent’ diseases which usually require the use of health services when the damage has become difficult to control. Such is the case of Type 2 diabetes and hypertension, where more than half of cases are diagnosed when complications appear [16].

Recent proposals for risk and health damage prevention programs with emphasis on early detection are being developed in several regions of Mexico. These proposals, in addition to developing various activities for detection and prevention of risks in diabetes and hypertension, are mainly aimed at the pre-diabetic or pre-hypertensive population.

Another important component of these proposals is that, besides involving traditional health professionals (physicians and nurses), psychologists, sociologists and anthropologists are also included as part of the health team that develops such actions. Involving social science professionals in these health care programs is extremely important if we consider the cultural and social aspects that determine the behavior of the user with a chronic disease, who demands services.

Increased coverage by schemes of ‘Universal Health Insurance’ has not been sufficient to meet the challenges of chronic health problems in LACs [17]. In terms of changes in the health system, a change is needed from the current system of care which is based on a biomedical, curative, fragmented and inequitable model towards a socio-medical model that is preventive, comprehensive and equitable.

The idea of a socio-medical model highlights the role of social determinants and includes social science professionals as part of the health team; it has a holistic approach and perspective. It includes the need to identify the impact of social determinants as risk factors, and also proposes the incorporation of content on social determinants, prevention and detection in the training of doctors and nurses.

Certainly, the holistic component refers to the design, development and allocation of resources for activities aimed at key stages of the natural history of the disease: detection, prevention, cure and rehabilitation, and to stop focusing only on healing and rehabilitation. This will enable more effective detection and control of chronic illness, with a consequent decrease in the effects of complications and treatment noncompliance. In most LACs, out of 100 people with diabetes, only 50 are diagnosed, and of these 50 only 30 continue with treatment and remain in control. Such patients have shown evidence of increased knowledge of risk factors and take on preventive actions to avoid complications once they are diagnosed with diabetes [18]. Certainly, the proposed approach will allow for better control of diagnosed cases, involving patients in self-care and a greater understanding of the consequences of failing to prevent the complications of diabetes.

More resources need to be allocated to design, implement and monitor strategies to move from an approach of treating, to one of preventing diabetes. In all LACs, there is little or no intervention for the pre-diabetic population. We need to develop and validate new methods of evaluating the epidemiological and economic burden in terms of direct costs (health care) and indirect costs (temporary disability, permanent disability and premature mortality).

In all LACs, it is necessary to implement new models of care and health management that can respond to the diversification and quantity of health services that will be needed as a result of the epidemiological transition, particularly for diabetic and hypertensive patients.

### Actions to ensure effective diabetes management in Latin America

The current model of care must go through a detailed review in order to propose changes in the physical infrastructure of facilities and the training of health personnel. The ways in which this can be done are outlined below:

- Develop infrastructure to expand screening programs for higher levels of detection, prevention and control. We also have to implement changes in the continuing education programs for health personnel to enable a greater focus on primary care and preventive medicine (mainly for doctors and nurses). Social science professionals should be integrated into health services (medical anthropologists and medical sociologists) to form part of the health team to implement new strategies for detection, prevention and control of chronic diseases (particularly in countries or areas with high indigenous populations with Type 2 diabetes).
- Implement a research line on trends in demand and costs for diabetes and develop new financing schemes with greater allocation of resources to new programs targeted to screening and prevention in the pre-diabetic population.
- Design and implement monitoring systems of epidemiological surveillance (number of cases) and economic burden for a periodic measurement that allows for assessment (preferably annual or biannual) of the impact of new strategies in epidemiological trends, as indicators of direct and indirect costs. This action will help keep track of the number of identified cases and the likely cost of those to the health care system, with a view to refining funding models and ensuring resources are going where they are needed.
- Design patterns of resource allocation to ensure that financial requirements are met to address diabetes based on expected demand. These patterns must integrate indicators of clinical perspective (inpatient and outpatient number of cases), epidemiological perspective (number of cases expected in the short term), organizational perspective (number of cases to be taken care of, by level of care) and economic perspective (average cost case of management by level of care).
- Implement monitoring systems accompanied by cost-containment strategies for cost of weight-by-cost items. For example, knowing that in case management the cost of medicines is high, it will be necessary for each institution to review its agreements with the pharmaceutical industry on the purchase of medicines for diabetes.
- In order to encourage people to avoid Type 2 diabetes, knowledge of the relative weight of the management of diabetes based on the annual family income, as well as required knowledge of the cost of complications to the users, should be made available through a bulletin sent to patients and their relatives, and to the community as a whole. Also, a list of recommendations is needed to promote greater self-care, monitoring of risk factors and the benefits of carrying out these measures and, more importantly, to avoid falling into a catastrophic situation (to avoid an impact of >30% of the family income) because of diabetes costs.
- With regard to the indirect costs to society due to loss of productivity from premature mortality, and temporary or permanent disability attributable to diabetes, companies must establish programs in the workplace for increased detection, prevention, treatment and control of diabetes and its complications. This will require developing agreements with the health system and workers and will have a positive impact on competitiveness and productivity.
- The various universal health insurance schemes that LACs have implemented have already provided the benefits of increased coverage, in terms of population size and in health needs not previously served by the health system, especially in maternal and child health, some chronic diseases, as well as some emerging diseases (AIDS, malaria, and so on.). Even with such benefits, limitations persist in the current structure of the health systems. Indeed, this structure has been the same for the past half century. The health systems of these countries must be reorganized to be more consistent with the state of the art of medicine and with the population’s health needs.

## Summary

In the context of ongoing reforms in the health systems of LACs, and taking into account the state of the art of medicine, universal coverage for health care will be effective only if it meets the health needs in all phases of the natural history of disease: prevention, detection, treatment and rehabilitation, particularly in the case of chronic health problems.

Certainly, the challenges posed in this paper are based on the evidence that universal coverage to address diabetes poses great challenges for detection and prevention. Not only does the health system not solve half of the diabetes cases, but it does little or nothing to detect the population with pre-diabetes.

We need to emphasize that, although medicine provides all the elements to solve the problem of diabetes, the health system is limiting its services to the patient population that requires healing or rehabilitation from complications.

Indeed, as health systems are organized in the LACs, focusing only on cure and rehabilitation, the cases that need detection and prevention of diabetes are not knocking on the door of the health system. We currently ignore the pre-diabetic population. This is even more serious if we consider that a large percentage of people with diabetes request the services of the health system when they already have some of the major complications in co-morbidity with diabetes: nephropathy, retinopathy, neuropathy and cardiovascular disease.

In other words, the absence of a culture of health behavior based on a preventive medicine model to meet the challenges of chronic diseases, such as diabetes, further complicates the situation in a health system that from its inception was designed, structured and organized to respond basically to communicable diseases and focus on curing or rehabilitating the sick person.

All of the above, in economic terms has important implications for medicine and health care, for families and patients with diabetes and for society as a whole. These implications represent two important economic burdens: the direct costs of detecting, preventing, treating and rehabilitating patients with diabetes or pre-diabetes; and indirect costs for temporary disability, permanent disability and premature death from complications of diabetes.

The panorama of challenges facing countries undergoing an epidemiological transition, including Type 2 diabetes and other chronic diseases, is not easy to face and solve, even with recent health care reforms implemented in LACs. If these schemes do not develop effective strategies for universal coverage, with changes in the model of care and more resources allocated to detection and prevention of chronic illness, the problem will continue. A transition from a curative model to a more holistic model that emphasizes prevention, detection, and long-term follow-up is urgently required. In the absence of these changes, chronic illnesses will continue to be poorly detected and universal health coverage will not be effective.

### Box 1 Current trends and challenges to be faced the coming years by the LACs

- Worldwide, Type 2 diabetes has increased alarmingly in recent years, and is projected to continue to rise.
- In LACs, health systems continue with the same model of care that was integrated since the 1940s and have not yet solved the challenges of treating Type 2 diabetes and its complications.
- In the context of the epidemiological transition, diabetes could cause a financial collapse of the health system and household economies. Currently in LACs, on average 25% of health expenditure is related to treating diabetes and related complications.
- If the current trend continues, there will be catastrophic expenditures for the health system and families - out of 100 pesos spent on diabetes in LACs, 53 are funded by households and 47 by health institutions.
- Failure to implement key changes in the health system will present a high cost to society due to premature mortality and increased disabilities caused by Type 2 diabetes.
- Health systems should move from an approach of treating diabetes to one of preventing diabetes.
